# Pulsatilla decoction alleviates DSS-induced UC by activating FXR-ASBT pathways to ameliorate disordered bile acids homeostasis

**DOI:** 10.3389/fphar.2024.1399829

**Published:** 2024-06-21

**Authors:** Ying Xiao, Ya-qian Jia, Wen-juan Liu, Chun Niu, Zhan-hai Mai, Jia-qi Dong, Xiao-song Zhang, Zi-wen Yuan, Peng Ji, Yan-ming Wei, Yong-li Hua

**Affiliations:** College of Veterinary Medicine, Institute of Traditional Chinese Veterinary Medicine, Gansu Agricultural University, Lanzhou, China

**Keywords:** Pulsatilla decoction, ulcerative colitis, PCR array, bile acid receptor, FXR, ASBT

## Abstract

**Ethnopharmacological relevance:** Pulsatilla decoction (PD) is a classical prescription for the treatment of ulcerative colitis. Previous studies have demonstrated that the therapeutic efficacy of PD is closely associated with the activation of Farnesoid X receptor (FXR). The activity of FXR is regulated by apical sodium-dependent bile acid transporter (ASBT), and the FXR-ASBT cascade reaction, centered around bile acid receptor FXR, plays a pivotal role in maintaining bile acid metabolic homeostasis to prevent the occurrence and progression of ulcerative colitis (UC).

**Aim of the study:** To elucidate the underlying mechanism by which PD exerts its proteactive effects against Dextran Sulfate Sodium Salt (DSS)-induced ulcerative colitis, focusing on the modulation of FXR and ASBT.

**Materials and methods:** To establish a model of acute ulcerative colitis, BALB/C mice were administered 3.5% DSS in their drinking water for consecutive 7 days. The disease activity index (DAI) was employed to evaluate the clinical symptoms exhibited by each group of mice. Goblet cell expression in colon tissue was assessed using glycogen schiff periodic acid-Schiff (PAS) and alcian blue staining techniques. Inflammatory cytokine expression in serum and colonic tissues was examined through enzyme-linked immunosorbent assay (ELISA). A PCR Array chip was utilized to screen 88 differential genes associated with the FXR-ASBT pathway in UC treatment with PD. Western blotting (WB) analysis was performed to detect protein expression levels of differentially expressed genes in mouse colon tissue.

**Results:** The PD treatment effectively reduced the Disease Activity Index (DAI) score and mitigated colon histopathological damage, while also restoring weight and colon length. Furthermore, it significantly alleviated the severity of ulcerative colitis (UC), regulated inflammation, modulated goblet cell numbers, and restored bile acid balance. Additionally, a PCR Array analysis identified 21 differentially expressed genes involved in the FXR-ASBT pathway. Western blot results demonstrated significant restoration of FXR, GPBAR1, CYP7A1, and FGF15 protein expression levels following PD treatment; moreover, there was an observed tendency towards increased expression levels of ABCB11 and RXRα.

**Conclusion:** The therapeutic efficacy of PD in UC mice is notable, potentially attributed to its modulation of bile acid homeostasis, enhancement of gut barrier function, and attenuation of intestinal inflammation.

## 1 Introduction

Pulsatilla decoction (PD) is derived from Zhang Zhong Jing’s Treatise on Typhoid, which states that PD is the mainstay for individuals with excessive heat and profit. The complete formula consists of Pulsatilla chinensis (Bunge) Regel (Bai Tou Weng), Phellodendron chinense C.K.Schneid. (Huang Bai), Coptis chinensis Franch. [Ranunculaceae] (Huang Lian), and Fraxinus chinensis Roxb. [Oleaceae] (Qin Pi). These four herbs are combined to detoxify heat and cool blood in order to alleviate dysentery symptoms (Liu et al., 2020; Liu et al., 2022). Due to its remarkable anti-inflammatory properties, PD is extensively employed in the treatment of Ulcerative colitis (UC) (Liu et al., 2021). PD suppresses intestinal inflammation, with a marked decrease in colonic infiltration of innate immune cells, through decreasing MMP-7 expression (Lin et al., 2022). PD may repair UC by maintaining the homeostasis and diversity of intestinal flora, increasing the content of SCFAs, and repairing the colonic mucosal barrier (Niu et al., 2023). PD has a good therapeutic effect on UC mouse model, and its potential mechanism may be related to the activation of PI3KAktmTORC1 signaling pathway (Wang et al., 2022).PD exhibits various pharmacological activities including antibacterial, antidiarrheal, anti-inflammatory, anti-ulcer, and immune regulation effects (hong Li et al., 2020). Our research team utilized a targeted metabolomics approach to investigate the impact of PD on UC. Significant alterations were observed in bile acids levels within serum, colon, bile, feces, and liver samples as well as in the expression of Farnesoid X receptor (FXR), a bile acid receptor found in liver and intestine tissues. These findings suggest that PD modulates the bile acid metabolism pathway by activating intestinal FXR (li Hua et al., 2021). However, the precise signaling mechanism through which PD regulates FXR involved in inflammatory bowel disease (IBD) therapy remains unclear. Additionally, it remains unknown how upstream and downstream genes related to FXR and ASBT change during PD treatment of IBD.

UC is a non-specific colonic inflammation of unknown etiology, characterized by severe diarrhea, weight loss, and bloody stool [908]. It is widely prevalent worldwide and is related to host genetics, immunity, inflammatory response, microbiota, metabolic disorders and other factors (Axelrad et al., 2021). Given the side effects, toxicity, tolerance issues and high cost of conventional drugs for UC treatment (Sandborn et al., 2020), there is an urgent need to discover novel therapeutic agents and strategies. Research has demonstrated that bile acid (BAs) metabolic disorders play a pivotal role in the pathogenesis of UC patients (Sinha et al., 2020). Bile acids not only function as digestive surfactants but also serve as crucial cell signaling molecules that activate multiple signaling pathways involved in regulating various vital biological processes (Ding et al., 2015). The bile acid-activated nuclear receptor FXR plays a central role in modulating bile acid and lipid metabolism as well as regulating inflammatory responses and barrier function. FXR participates in the regulation of bile acid homeostasis along with intestinal barrier restoration during the pathophysiological progression of diverse gastrointestinal diseases such as IBD, colorectal cancer, and type 2 diabetes (Wang et al., 2018). Given that FXR and bile acids (BAs) play crucial roles in maintaining gastrointestinal homeostasis, any disruption in FXR expression or BAs composition may contribute to the development of gastrointestinal symptoms (Mosińska et al., 2018). In a mouse model of colitis, downregulation of FXR mRNA expression was observed in inflammation of the colonic mucosa (Zhao et al., 2020). Mencarelli et al. demonstrated that FXR−/−mice exhibited mild to moderate cell infiltration and increased expression of pro-inflammatory cytokines in the colonic mucosa compared with wild-type mice (Vavassori et al., 2009; Hegyi et al., 1983). Therefore, understanding how changes in intestinal luminal bile acid levels impact intestinal function under normal and inflammatory conditions ultimately relies on the intricate interplay between pro- and anti-inflammatory mediators released by epithelial cells (Hegyi et al., 2021) Notably, naïve FXR^−/−^ mice displayed intestinal inflammation characterized by cellular swelling within the colonic mucosa and elevated expression of pro-inflammatory cytokines when compared to wild-type mice. Dysregulation of intestinal ecology is considered one of the primary predisposing factors for UC, as it leads to dysbiosis. Activation of TGR5 on intestinal epithelial cells can stimulate synthesis and release of IL10 by anti-inflammatory cells as well as promote activation of Treg cells (Yang et al., 2022). Recent findings have indicated that activating FXR may alleviate colitis symptoms. Furthermore, through reducing goblet cell loss, FXR can inhibit intestinal inflammation and protect the integrity of the intestinal barrier (Fiorucci et al., 2021a). In the ileum, activation of FXR depends on enterocyte-mediated uptake facilitated by Apical sodium-dependent bile acid transporter protein (ASBT) (Fiorucci et al., 2021b). By inhibiting ASBT expression, activated FXR reduces bile acid uptake (Fiorucci et al., 2021a). However, the activity of bile acid receptor FXR is affected by ASBT, and the absorption of bile acid in ileum is mediated by ASBT, which affects FXR activity. Therefore, the metabolic balance between bile acid and gut microbiota in the FXR-ASBT cascade reaction with bile acid receptor FXR as the core is crucial to prevent the occurrence and development of inflammation. Consequently, further investigation is warranted to elucidate how PD regulates upstream and downstream genes involved in regulating ASBT-FXR.

Therefore, this study aims to investigate the potential mechanism of PD in alleviating UC based on the FXR-ASBT signaling pathway for the first time using a 3.5% DSS-induced UC mice model, thereby providing a scientific foundation for the clinical application of PD in UC treatment.

## 2 Materials

### 2.1 Pulsatilla decoction ingredients

Pulsatilla decoction composed of *Pulsatilla chinensis* (Bunge) Regel (Bai Tou Weng), *Phellodendron chinense* C.K.Schneid. (Huang Bai), *Coptis chinensis* Franch. [Ranunculaceae] (Huang Lian) and *Fraxinus chinensis* Roxb. [Oleaceae] (Qin Pi). It was purchased from the wholesale market of Yellow River medicinal materials in Lanzhou, and was identified by the Department of Traditional Chinese Veterinary Medicine, College of Animal Medicine, Gansu Agricultural University. Pulsatilla decoction compound Chinese medicine origin information is shown in Table 1.

**TABLE 1:** The medicinal formula of PD.

Latin name (Chinese name)	Medicinal parts	Place of production	Voucher numbers	Storage location
*Pulsatilla chinensis* (Bunge) Regel	Root	Jilin	GSAUTCM-20210901	Chinese Veterinary Laboratory, College of Veterinary Medicine, Gansu Agricultural University
*Phellodendron chinense* C.K.Schneid	Rhizome	Sichuan	GSAUTCM-20210902	
*Coptis chinensis* Franch. [Ranunculaceae]	Bark	Sichuan	GSAUTCM-20210903	
*Fraxinus chinensis* Roxb. [Oleaceae]	Bark	Hebei	GSAUTCM-20210904	

### 2.2 Reagents

Salazosulfapyridine (SASP) enteric-coated tablets, approved by H31020557, purchased from Shanghai Xinyi Tianping Pharmaceutical Co; Dextran Sodium Sulfate (DSS) (MW: 36000–50000) was purchased from Reed Biotechnology Co.

### 2.3 Animals

Male BALB/C mice (8 weeks old, 18–20 g) were purchased from the Experimental Animal Center of Lanzhou Veterinary Research Institute, Chinese Academy of Agricultural Sciences, Animal License No.SCXK (Gan) 2015-0001. Animals were purchased for adaptive feeding for 7 days to start the experiment. The mice were fed at a temperature of about 25°C, a humidity of about 50%, and a dark cycle. Full-price compound feed and pure water for free intake in mice. Animal welfare and experimental procedures conformed to the Guide for the Care and Use of Laboratory Animals (Ministry of Science and Technology, China, 2006) and were approved by the animal ethics committee and institutional animal care (license approval number: GAU-LC-2021-009).

## 3 Methods

### 3.1 Preparation of Pulsatilla decoction

*Pulsatilla chinensis* (Bunge) Regel (Bai Tou Weng), *P. chinense* C.K.Schneid. (Huang Bai), *C. chinensis* Franch. [Ranunculaceae] (Huang Lian) and *F. chinensis* Roxb. [Oleaceae] (Qin Pi) (20, 15, 10, and 20 g respectively) were referred to the prescription ratio of PD recorded in the 2015 edition of ' Chinese Veterinary Pharmacopoeia '. The herbs were soaked in distilled water for 1 hour, followed by multiple rounds of extraction using increasing amounts of water and heating reflux method. The resulting filtrate was concentrated to obtain a decoction with a concentration of 1 g/mL at 60°C, which was then stored at 4°C for future use. In the initial stage of our research, HPLC analysis was conducted to determine the contents of berberine, anemoside B4, jatrorrhizine hydrochloride, palmatine hydrochloride, aesculin, and aesculetin in Pulsatilla decoction. The respective content percentages were found to be 8.26%, 4.71%, 0.12%, 0.38%, 0.7%, and 0.36% (li Hua et al., 2019). (Near Supplementary Material).

### 3.2 Establishment of DSS-induced UC mice model and PD treatment

Adaptive feeding for 1 week before modeling. Ninety BALB/C mice were randomly divided into control group (CON), model group (3.5% wt/vol DSS, DSS), positive control group (0.45 g/kg salazosulfapyridine, SASP), Pulsatilla high-dose treatment group (10.8 g/kg Pulsatilla decoction, PDHT), Pulsatilla medium-dose treatment group (5.4 g/kg Pulsatilla decoction, PDMT), Pulsatilla low-dose treatment group (2.7 g/kg Pulsatilla decoction, PDLT), (n = 15). The control group was given normal saline, and the other groups were given 3.5% DSS drinking water to BALB/C mice for 7 days to establish an acute UC model. At the same time of modeling, different doses of Pulsatilla decoction were administered daily for treatment. Sulfasalazine was used as positive control drug. Fresh DSS aqueous solution was replaced every morning, and the volume of mice was 0.015 g/mL.

### 3.3 DAI score

During the experiment, the body weight loss, fecal traits and degree of hematochezia of mice in each group were recorded every day. The criteria for judging the DAI were as described in the literature (Yuan et al., 2019).

### 3.4 Histological evaluation

The colon length and histological damage were used to evaluate the treatment of UC in mice and whether the UC model was successfully constructed. Colon tissues were removed, fixed in 10% formaldehyde solution, followed by routine paraffin embedding, sectioning, dewaxing, HE staining, and post-photography (Leica DFC microscopic photography system) to observe colon tissue lesions. Histological scoring according to accepted methods (Yuan et al., 2019).

### 3.5 Detection of goblet cells

The number of goblet cells after Alcian blue (AB) staining was determined by observing the colon tissue of mice under light microscope. The glycogen deposition in tissues was determined by schiff periodic acid shiff (PAS) staining.

### 3.6 Measurement of cytokines in serum and colon tissue

According to the enzyme-linked immunosorbent assay (ELISA) kits (Neobioscience Technology, Shanghai, China) instructions, the absorbance of cytokines was detected at 450 nm with a Microplate reader 160(Spectra Max i3x, Molecular Device, United States). The levels of IL-1β(EMC001b.96), IL-10 (EMC005.96), IL-17 (EMC008.96) and IL-23 (EMC114.96) in serum and colon groups were calculated according to the standard curve.

### 3.7 Measurement of total bile acid and total cholesterol

The levels of serum were quantified strictly according to Total cholesterol TCH kits (Item No. A11111) and total bile acid TBA kits (Item No. E00321) (Nanjing Jiancheng Bioengineering Institute, Nanjing, China) in all groups of mice.

### 3.8 The PCR array chip was used to identify differentially expressed genes associated with the FXR-ASBT pathway

RNA quality control using an RT2 RNA QC PCR Array. The RT2 RNA QC PCR Array is designed to assess the quality of 12 RNA samples simultaneously before gene expression analysis using RT2 Profiler PCR Arrays. Excise the tissue sample from the animal or remove it from storage. Remove RNA protect stabilized tissues from the reagent using forceps. Determine the amount of tissue. RNA extraction using RNeasy Mini Kit (50), (item no. 74104).

Purified RNA was prepared into cDNA using the RT2 First Strand Kit (12), (item no. 330401). The cDNA was added to RT2 SYBR Green ROX qPCR Mastermix (12), (item no. 330502), and the mixed reactants were added to each hole of RT2 custom pcr array (96-12), (item no. 330171). It is necessary to ensure that the amount of each hole is accurate, centrifuge, react with real-time PCR instrument, export data for statistical analysis, and use 2^−ΔΔCT^ formula to calculate the quantitative analysis of target gene expression. Export the CT values for all wells to a blank Excel spreadsheet. Data analysis can then be conducted using a spreadsheet-based tool that can be downloaded from a QIAGEN website.

### 3.9 Western blot analysis

The anterior colon tissue of each group of mice was taken and weighed (about 0.025 0 g), and then the cut tissue was added with RIPA lysis buffer (Solarbio, Beijing, China) and two iron beads. Then it was put into a frozen high-throughput tissue grinder to fully lyse. This process was operated on ice to extract protein. The protein concentration was determined by BCA kit (Solarbio, Beijing, China, pc0020). The protein was separated by 10% SDS-PAGE and transferred to 0.22 μm PVDF (Millipore, MA, United States) membrane after electrophoresis for 1 h and 30 min. After the membrane was transferred, it was sealed at room temperature for 2 h in 5% (wt/vol) non-fat milk powder, then the membrane was incubated overnight at 4°C with primary antibody against primary antibody Rabbit Polyclonal antibody diluted with 5% (wt/vol) non-fat milk powder. Beta-actin (Proteintech, 20536-1-AP), FXR/NR1H4 (Bioss, bs-12867R), CYP7A1 (Bioss,bs-21430R), ASBT/SLC10A2 (Bioss, bs-23146R), CYP27A1 (Bioss, bs-5049R), ABCB11(Ab-AF7593), RXRα (Bioss, bs-20769R), TGR5/GPBAR1 (CSB-PA819471LA01HU), Fgf15 (CSB-PA522052LA01MO). After incubation, the membrane was washed with TBST buffer (5 min once, 8-10 times), added with HRP-conjugated Affinipure Goat Anti-Rabbit IgG (H + L) (Proteintech, SA00001-2), and incubated at room temperature for 1 h. The ECL chemiluminescence liquid was added, and the chemiluminescence signal was collected and photographed on the Amersham Imager 600 chemiluminescence instrument (GE Healthcare Bio-Sciences AB, Sweden), and the pictures were saved. ImageJ software was used to analyze the gray value of each group of pictures.

### 3.10 Statistical analyzes

All data in this paper were analyzed by SPASS 25.0 and GraphPad Prism 8.0.2 software. Statistical differences between groups were compared using one-way ANOVA (oneway ANOVN). The data were expressed as mean ± standard error (mean ± SEM), and *p* < 0.05 was considered statistically significant.

## 4 Results

### 4.1 PD alleviates the symptoms in UC mice

**FIGURE 1:**
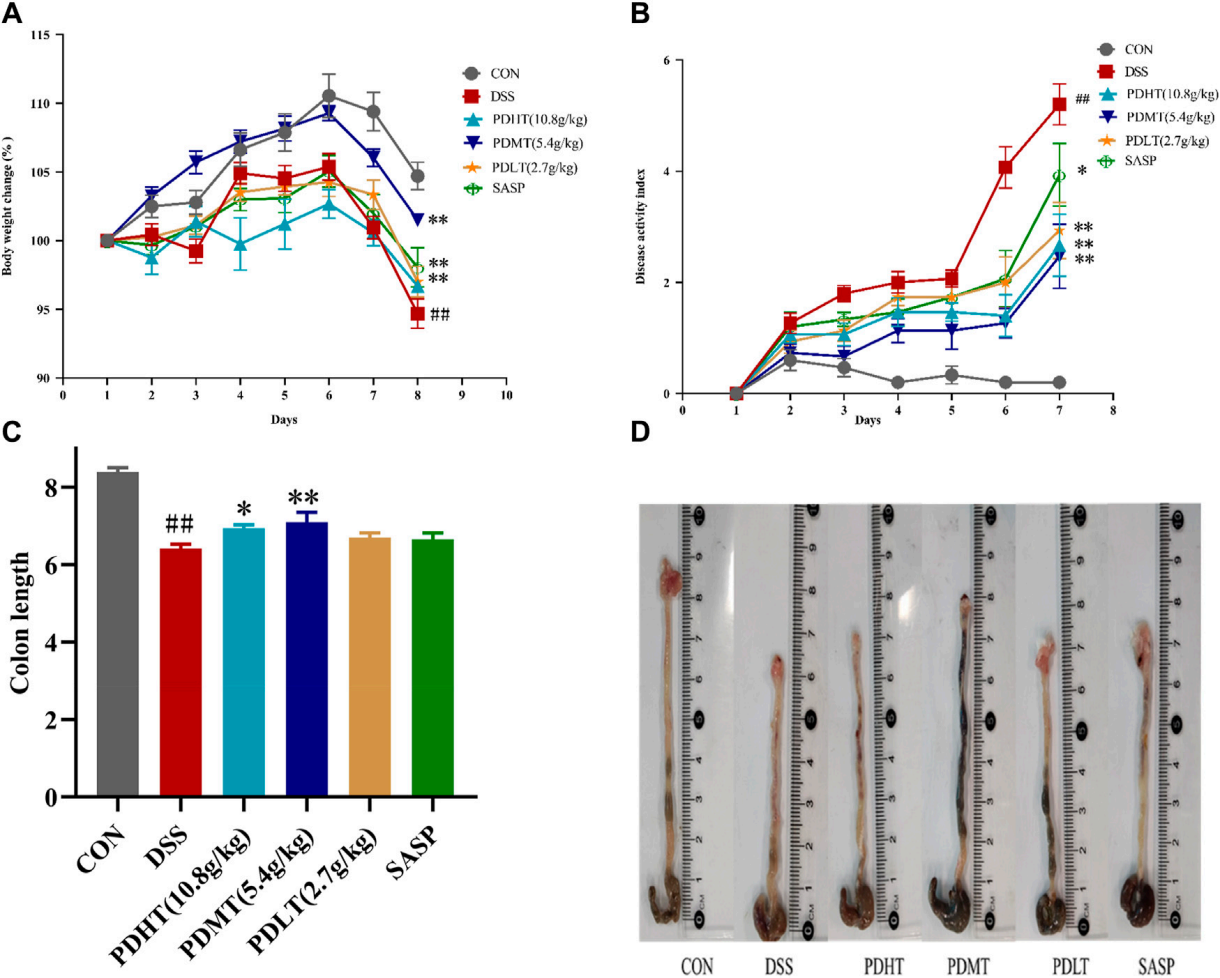
Effect of PD on clinical symptoms of DSS-induced UC mice. **(A)** Daily changes of weight change percentage in different groups; **(B)** Daily disease activity index changes; **(C)** Colon length of mice in each group; **(D)** Macroscopic observation of mice colon. Data are shown as mean ± SEM (*n* = 15), #*p* < 0.05, ##*p* < 0.01 Compared with CON, *p < 0.05, **p < 0.01 Compared with DSS.

The DSS-induced acute ulcerative colitis model in mice was successfully replicated, exhibiting prominent clinical manifestations including hematochezia, diarrhea, weight loss, and colon shortening. On the third day post-DSS administration, the mice exhibited soft feces with a slight amount of bleeding. By the sixth day of modeling, severe hematochezia occurred in all groups except for the CON group, accompanied by declining body weight and increasing Disease Activity Index (DAI). PD demonstrated a certain inhibitory effect on weight loss and DAI elevation. Starting from the seventh day, compared to the CON group, the DSS group continued to experience weight loss while PD at each dosage and SASP effectively mitigated body mass reduction (Figure 1A). The results indicated that DAI increased and colon length decreased in the DSS group compared to the CON group; however, both PD and SASP reduced DAI scores (Figure 1B) and restored colon length in mice (Figures 1C, D). Among them, PD exhibited superior therapeutic effects on UC mice with optimal efficacy observed at a dose of 5.4 g/kg; thus PDMT group was selected for subsequent experimental studies.

### 4.2 PD alleviates pathological injury in UC mice

**FIGURE 2:**
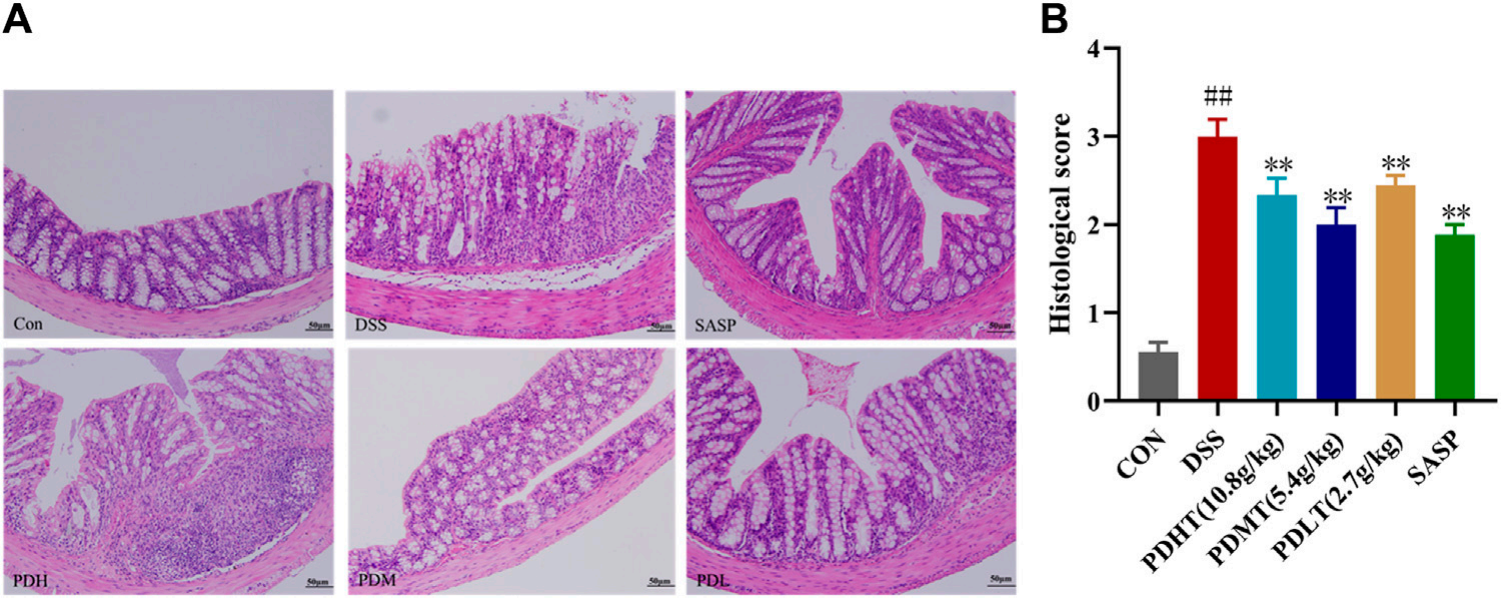
Effect of PD on colon histopathology in DSS-induced UC mice. **(A)** Representative images of colon tissue in each group stained with hematoxylin and eosin (H&E)-stained (200×). **(B)** Statistics of colon histopathology scores. Data are shown as mean ± SEM (*n* = 3), #*p* < 0.05, ##*p* < 0.01 Compared with CON, *p < 0.05, **p < 0.01 Compared with DSS.

The pathological changes of colon tissue in mice were observed using HE staining. In the CON group, intact colonoscopic structure, well-arranged intestinal villi, complete intestinal crypts, abundant goblet cells, absence of inflammation and histological abnormalities were observed. In the DSS group, there was detachment of intestinal villi with blurred structure, missing intestinal crypts and dissolved intestinal glands. Additionally, there was presence of inflammatory cell infiltration, local lesions and intestinal epithelial hyperplasia. Both PD and SASP showed varying degrees of improvement in histological changes caused by inflammation as evidenced by restoration of intestinal villi structure, enhanced secretory function of intestinal glands and reduction in inflammatory cell absorption. All doses of PD exhibited favorable effects (Figure 2A), with PDMT demonstrating a superior histological score (Figure 2B).

### 4.3 PD increased the number of goblet cells in UC mice

**FIGURE 3:**
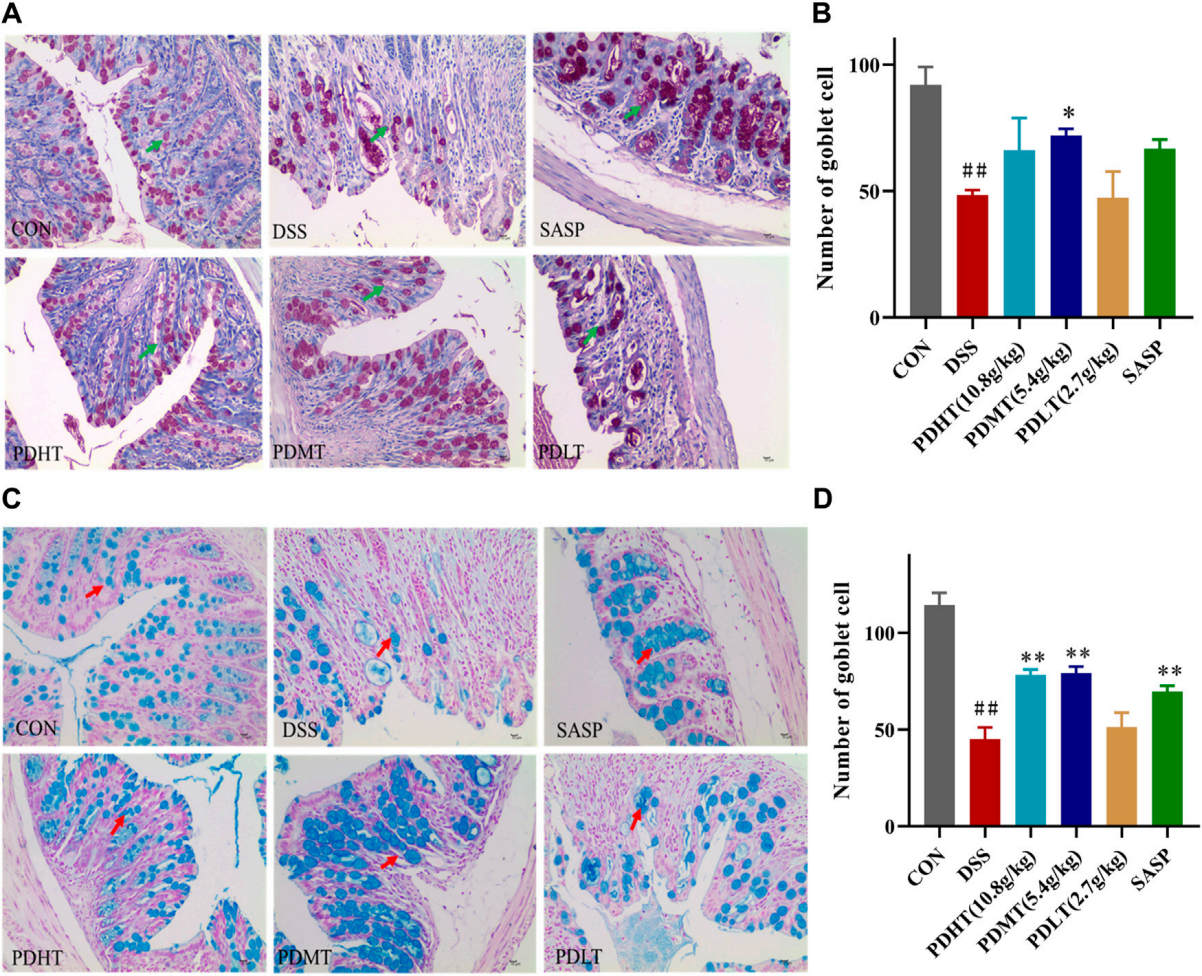
AB-PAS staining of colon tissue **(A)** PAS staining of colon tissue (400×). **(C)** Alcian Blue of colon tissue (400×). **(B, D)** Statistics of the number of goblet cells. Data are shown as mean ± SEM (*n* = 3), #*p* < 0.05, ##*p* < 0.01 Compared with CON, *p < 0.05, **p < 0.01 Compared with DSS.

The administration of PD resulted in an increase in the number of goblet cells in mice with UC. Goblet cell quantification was performed by examining colon tissue sections stained with periodic acid-Schiff (PAS) and Alcian blue (AB) under a light microscope. Goblet cells secrete mucus, as indicated by positive PAS staining (Figures 3A, C), which plays a crucial role in lubricating and protecting the intestinal epithelium. PAS and AB staining revealed a significant reduction in goblet cell count in the DSS group compared to the CON group; however, treatment with PD significantly restored goblet cell numbers (Figures 3B, D).

### 4.4 PD alleviates colonic inflammation in UC mice

**FIGURE 4:**
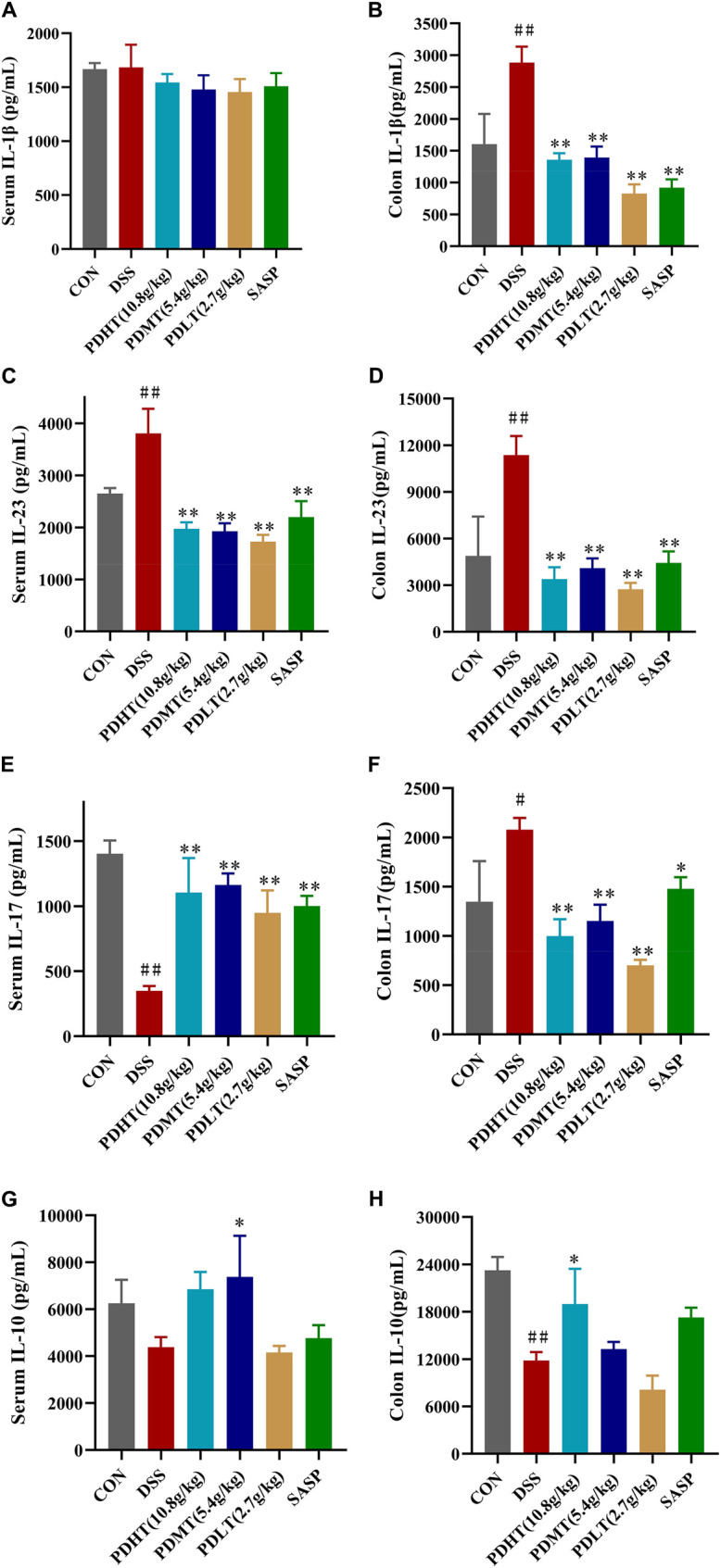
(Continued).

Compared with the CON group, the level of IL-10 in the colon of the DSS group was significantly reduced (Figures 4G, H), while the levels of IL-1β, IL-23, and IL-17 were significantly elevated (Figures 4A–F), indicating a pronounced inflammatory response in mice following induction of UC by DSS. In comparison to the DSS group, there was a certain increase in IL-10 levels in the PD group, suggesting that anti-inflammatory factors had a positive regulatory effect after treatment; meanwhile, there was a significant decrease in IL-23, IL-17 and IL-1β levels in the PD group. Moreover, the reduction of proinflammatory factors observed in the PD group was more prominent than that seen in the SASP group, demonstrating superior efficacy compared to SASP treatment. These findings indicate that PD treatment can effectively inhibit pro-inflammatory factors and suppress inflammation occurrence.

### 4.5 PD regulates serum total bile acid and cholesterol levels in UC mice

**FIGURE 5:**
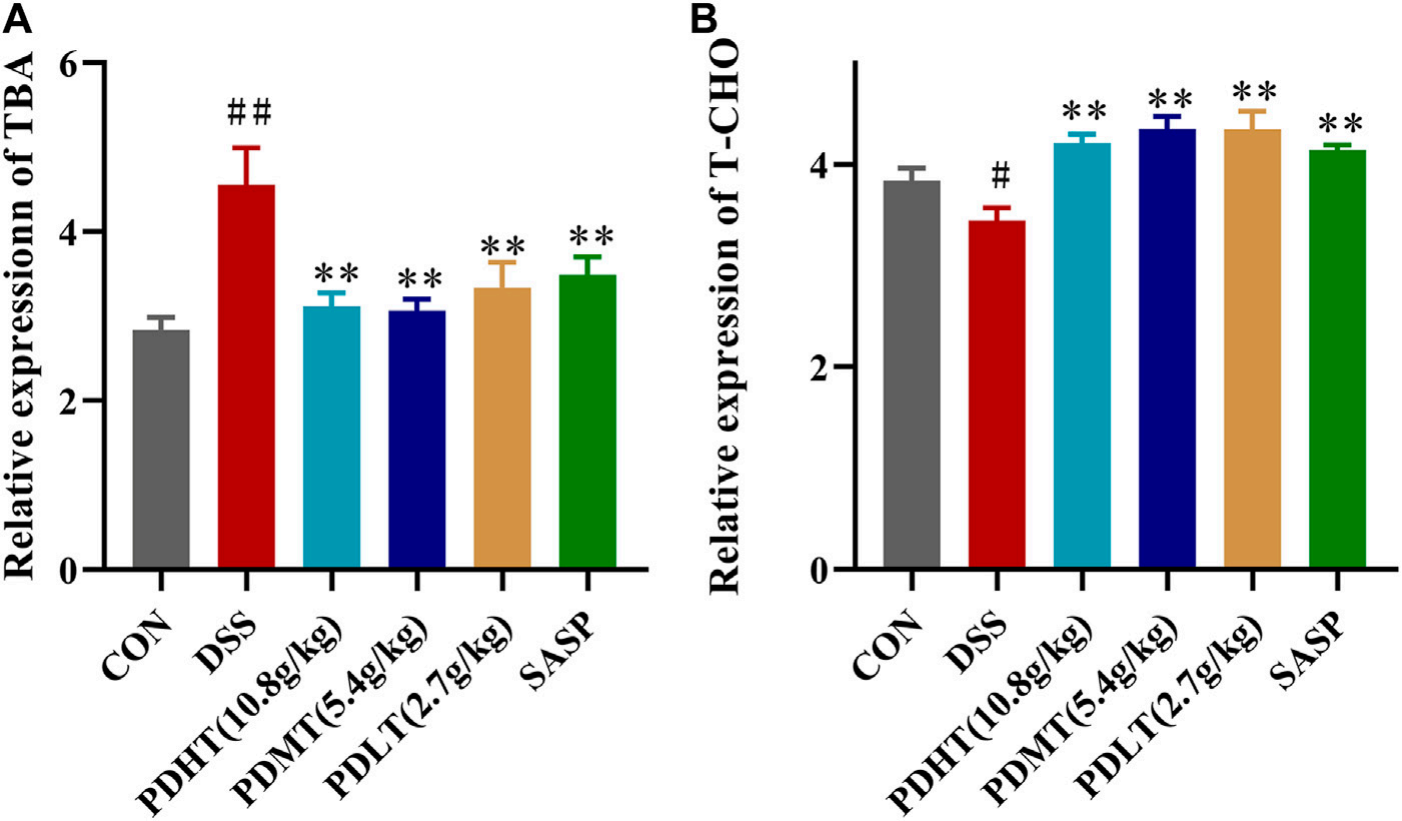
Effect of PD on the content of total bile acid and cholesterol in serum of DSS-induced UC mice. **(A)** Concentrations of TBA in serum. **(B)** Concentrations of T-CHO in serum. Data are shown as mean ± SEM (*n* = 8), #*p* < 0.05, ##*p* < 0.01 Compared with CON, *p < 0.05, **p < 0.01 Compared with DSS.

Compared with the CON group, the total bile acid content in the serum of mice in the DSS group was significantly increased and the total cholesterol content was significantly decreased, and the content was significantly decreased after treatment with PD and SASP (Figure 5A), but the treatment effect of PD group was better. There was no statistical difference, while the cholesterol content of PD and SASP group increased significantly (Figure 5B).

### 4.6 Screening of differential genes of FXR-ASBT pathway for the therapeutic effect of PD on UC mice

**FIGURE 6:**
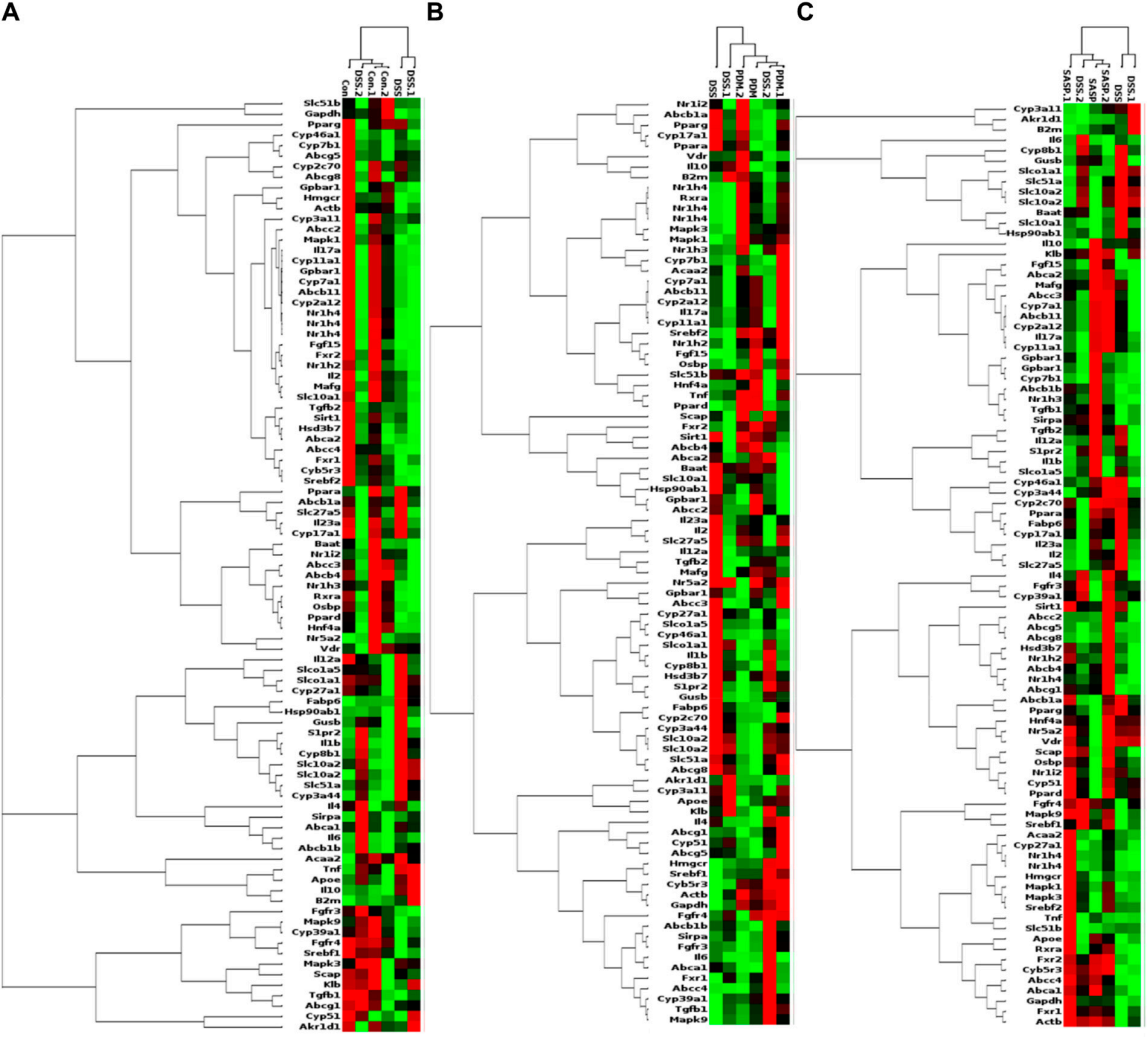
PCR array gene expression heat map. Note: Changes in gene expression caused by the treatment of DSS-induced UC with PD. The expression of 88 genes involved in the gut-liver axis of bile acid receptor in UC disease has been profifiled. Clustergrams create a heat map with dendograms to indicate which genes are coregulated. Redness and greenness represent the high and low expression of the gene, while blackness represents the equal expression of the gene. **(A)** DSS vs CON, **(B)** PDMT vs DSS, and **(C)** SASP vs DSS groups. *n* = 3 mice/group.

**FIGURE 7:**
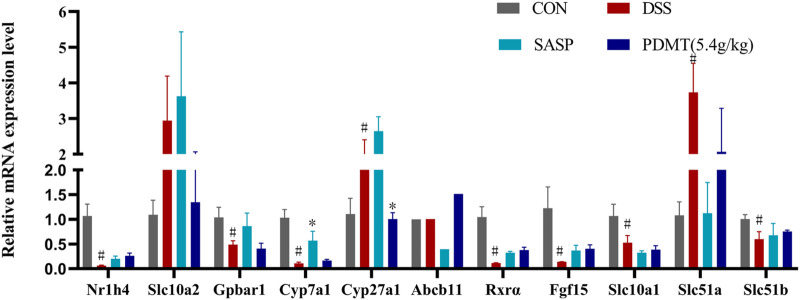
The changes of mRNA relative expression about FXR-ASBT pathway key gene. Relative expression of Nr1h4, Slc10a2, Gpbar1, Cyp7a1, Cyp27a1, Abcb11. Rxrα. Fgf15, Slc10a1, Slc51a and Slc51b mRNA. Data are shown as mean ± SEM (n = 3), #*p* < 0.05, #*p* < 0.05, ##*p* < 0.01 Compared with CON, *p < 0.05, **p < 0.01 Compared with DSS.

As shown in Table 2, Compared with the CON group, 42 of 88 genes in the colon tissue of DSS group were significantly changed. Among these genes, were upregulated including Slc10a2, Tnf, IL10, IL6, IL1b, S1pr2, Apoe, Slc51a, Fabp6, Cyp8b1, Abcb1b, Cyp-3a44, Abca1, 29 genes were downregulated including Gpbar1, Nr1h4, IL23a, IL17a, Tgfb2, Cyp7a1, Abcb11, Rxrα, IL2, Fxr2, Mafg, Cyp2a12, Slc51b, Fgf15, Osbp, Baat, Cyp17a1, Slc10a1, Abcc2, Abcc3, Abcc4, Abcb4, Akr1d, Cyb5r3, Cyp3a11, Cyp11a1, Nr1h2, Ppard, Hnf4a. After treatment with PD, a total of 21 genes were significantly restored compared with the DSS group. Among these genes, eight genes were upregulated including Nr1h4, IL17a, Cyp7a1, Abcb11, Cyp2a12, Fgf15, Slc27a5, Rxrα, 13 genes were downregulated including Slc10a2, IL6, IL1b, Slco1a5, Slc51a, KIb, Slco1a1, Cyp27a1, Cyp8b1, Abcg8, Abcb1b, Cyp3a44, Abca1. The SASP group significantly changed 20 genes. Among these genes, 14 genes were upregulated including Gpbar1, Nr1h4, Tnf, Apoe, Rxrα, Fgf15, Cyp27a1, Cyp7b1, Abcc3, Abcc4, Abcg5, Abcg8, Abcb1b, Abcb4. There were six downregulated genes including Slc10a2, IL6, Slc51a, Slco1a1, Cyp8b1, and Akr1d1. Figure 6 shows clustergram analysis and heat map graphs of gene expression data. The changes of mRNA relative expression about FXR-ASBT pathway key gene (Figure 7). Compared with the CON group, the mRNA expression of Nr1h4, Gpbar1, Cyp7a1, Abcb11, Rxrα, Fgf15, Osbp, Slc10a1 in the DSS group decreased, and increased after PD intervention. Compared with the CON group, the mRNA expression of Slc10a2, Cyp27a1 and Slc10a5 in the DSS group increased, and decreased after PD intervention. The results showed that PD could regulate the key genes of FXR-ASBT pathway in the colon of UC mice and maintain the balance of bile acids in the body. The specific mechanism of action needs to be further explored.

**TABLE 2:** Gene expression analysis, encompassing the statistical significance (*p*-values) and magnitude of change (fold regulation) for all genes examined.

	CON VS.DSS			PDMT VS DSS			SASP VS DSS		
Symbol	*p*-value	Regulation	Comments	*p*-value	Regulation	Comments	*p*-value	Regulation	Comments
Slc10a2	**0.006023**	**−3.64**		0.059608	−1.87		0.056810	**−2.05**	
Slc10a2	**0.001856**	−1.85		**0.042516**	−1.95		**0.045719**	**−2.14**	
Gpbar1	**0.012491**	**2.84**		0.870027	−1.09	B	0.099014	**2.17**	B
Gpbar1	**0.013633**	**4.81**		0.616059	1.10	B	0.111520	**2.44**	B
Nr1h4	**0.004756**	**21.65**		**0.026920**	**4.56**		0.069905	**2.40**	
Nr1h4	**0.007129**	**21.33**		**0.019047**	**4.29**		0.130688	**3.52**	
Nr1h4	**0.004918**	**23.86**		**0.015324**	**4.82**		0.116574	**3.98**	
Tnf	**0.026033**	**−2.78**	A	**0.008114**	1.50		0.191174	1.98	
Il10	0.066534	−1.69	B	0.766859	1.06	B	0.578567	1.17	B
Il23a	0.453435	1.70	B	0.868293	1.32	B	0.891690	1.05	B
Il17a	**0.014830**	**4.33**		**0.034668**	1.62		0.118607	1.36	B
Il6	0.207989	**−2.96**	B	0.255204	**−2.19**	B	0.192734	**−3.50**	B
Il1b	**0.011878**	**−9.11**	A	**0.036499**	**−2.57**		0.798750	−1.41	
Il4	0.376737	−1.40	B	0.725074	−1.37	B	0.971274	1.00	B
Tgfb2	0.165008	1.89		0.442961	−1.17		0.594841	1.14	
Tgfb1	0.753760	1.10		0.923479	1.01		0.173720	1.55	
Sirpa	0.218820	−1.40		0.328816	−1.30		0.201032	1.42	
Cyp7a1	**0.014232**	**4.63**	C	**0.033024**	1.73	C	0.102220	1.45	C
Abcb11	**0.014232**	**4.63**	C	**0.033024**	1.73	C	0.102220	1.45	C
Il12a	0.652063	−1.37	B	0.789765	−1.02	B	0.811593	1.09	B
Il2	**0.032807**	**3.36**		0.497786	1.37	B	0.959766	1.10	B
Fxr2	**0.022802**	1.69		0.254182	1.12		**0.007706**	1.35	
Fxr1	0.088512	1.33		0.403163	−1.07		0.094994	1.20	
Mafg	**0.038951**	**2.26**		0.890652	−1.01		0.146531	1.39	
Sirt1	0.155979	1.40		0.784288	1.04		0.284037	1.15	
S1pr2	0.070848	−1.91	A	0.236220	−1.53		0.973544	−1.01	
Hsd3b7	0.242710	1.26		0.078548	−1.25		0.051546	1.31	
Slco1a5	0.344274	−1.34	B	0.104215	−1.64	B	0.925902	−1.24	B
Apoe	**0.009896**	**−2.04**		0.161632	−1.34		**0.014005**	**2.16**	
Mapk9	0.417040	1.16		0.799727	−1.02		0.510661	1.12	
Mapk3	0.525423	−1.22		0.123806	1.23		0.341468	1.22	
Rxra	**0.003591**	**11.82**		**0.008819**	**3.71**		**0.007908**	**3.67**	
Hmgcr	0.079947	1.54		0.741358	1.05		0.467106	1.22	
Scap	0.767598	1.03		0.938492	1.01		0.493540	1.10	
Cyp2a12	**0.014232**	**4.63**	C	**0.033024**	1.73	C	0.102220	1.45	C
Mapk1	**0.016040**	1.48		**0.032600**	1.21		0.202952	1.17	
Slc51a	**0.025655**	**−2.65**		0.489611	**−2.22**		0.066449	**−3.27**	
Slc51b	**0.039254**	**2.45**		0.083385	1.56		0.460947	1.41	
Fgfr4	0.190602	1.35		0.936815	−1.07		0.700895	1.11	
Klb	0.964270	−1.07	A	**0.045491**	**−2.31**	A	0.274464	1.29	A
Fgfr3	0.709713	1.14		0.444410	−1.27		0.751062	−1.26	
Fgf15	**0.045069**	**9.58**		**0.026330**	**3.11**		**0.041668**	**3.06**	
Osbp	**0.004296**	1.98		0.101600	1.30		0.588429	1.07	
Nr5a2	0.674053	1.07		0.583478	−1.10		0.995587	−1.16	
Baat	0.139877	**2.11**		0.384866	−1.55	A	0.120638	−1.59	A
Slc27a5	0.685876	1.29	B	0.286214	1.65	B	0.985693	1.11	B
Fabp6	0.134978	**−2.45**	A	0.502666	−1.27		0.680813	1.29	
Slco1a1	0.346446	−1.27	B	**0.001235**	**−3.40**		**0.000924**	**−4.05**	
Cyp46a1	0.442891	1.41	B	0.212924	−1.45	B	0.411200	1.30	B
Cyp51	0.781850	−1.02		0.705416	−1.12		0.727772	1.00	
Srebf1	0.188868	1.29		0.878948	1.04		0.645137	1.09	
Cyp27a1	0.282130	−1.43		0.059814	−1.70		0.152716	1.74	
Cyp17a1	0.387728	1.76	B	0.915002	1.18	B	0.807169	1.36	B
Cyp8b1	**0.029657**	**−2.60**		**0.035927**	**−2.46**		0.066340	−1.96	B
Cyp7b1	0.512672	1.16	A	0.193238	1.35		0.193937	**2.34**	
Cyp2c70	0.262583	1.59	B	0.764661	−1.12	B	0.215007	1.56	B
Slc10a1	**0.028321**	**2.76**		0.620750	−1.17		0.484263	−1.17	
Abcc2	**0.032109**	**3.08**		0.893111	−1.15	B	0.406458	1.35	B
Abcc3	**0.009050**	1.95		0.839970	1.04		**0.025867**	1.67	
Abcc4	0.262463	1.61		0.600716	−1.12		0.098255	1.72	
Abcg5	0.789856	−1.14	B	0.969653	−1.37	B	0.204627	**2.55**	A
Abcg8	0.486779	1.21	B	0.305717	**−2.35**	B	0.319396	1.81	B
Abcb1a	0.748013	1.27		0.978677	1.05		0.936349	1.01	
Abcb1b	**0.042740**	**−2.50**	A	0.151196	−1.69	A	0.118376	1.90	
Abcb4	**0.001498**	**2.74**		0.292547	1.35	B	0.050481	1.79	B
Acaa2	0.467573	−1.10		0.231182	1.18		0.306137	1.46	
Akr1d1	0.536434	1.78	B	0.395723	−1.42	B	0.310042	−1.79	B
Cyb5r3	0.073250	1.79		0.278828	1.24		**0.037151**	1.53	
Cyp3a11	0.062576	**2.35**	B	0.706500	−1.08	B	0.479898	−1.22	B
Cyp3a44	**0.005866**	**−13.18**	A	0.065607	**−4.07**	A	0.995383	−1.03	
Cyp11a1	**0.014977**	**4.28**		**0.036038**	1.60		0.125464	1.34	B
Cyp39a1	0.317846	1.20		0.977195	1.01		0.843504	−1.11	
Vdr	0.243022	1.29		0.373059	1.32		0.790540	−1.31	
Nr1h3	**0.029129**	1.35		0.457459	1.11		0.108032	1.40	
Nr1h2	**0.045652**	1.71		0.067234	1.18		0.098695	1.25	
Srebf2	0.068240	1.21		**0.019763**	1.12		0.397411	1.14	
Nr1i2	0.143972	1.33		0.440123	1.15		0.624308	1.08	
Ppara	0.914929	1.05		0.854549	−1.05		0.511269	1.24	
Pparg	0.155379	1.43		0.640922	1.14		0.710648	−1.17	
Ppard	**0.010832**	1.95		0.056865	1.52		0.674847	1.05	
Abca1	0.120861	−1.67		0.061171	−1.70		0.089977	1.54	
Abca2	0.200633	1.46		0.572650	−1.04		**0.049416**	1.38	
Hnf4a	**0.008345**	1.87		0.082053	1.20		0.877293	−1.09	
Abcg1	0.750088	−1.08		0.913808	−1.04		0.056140	1.40	
Actb	0.080156	1.59		0.196979	1.28		0.115708	1.35	
Gapdh	0.058843	1.35		0.168467	1.14		0.125081	1.30	
B2m	0.163037	−1.55		0.789934	1.11		0.543190	−1.15	
Hsp90ab1	0.296283	−1.17		0.312501	−1.25		0.129709	−1.34	
Gusb	0.358898	−1.19		0.214609	−1.29		0.464476	−1.13	

In statistically significant genes, the amount of crease adjustment is marked in bold. “A" means that in the control group or detection group, the average threshold time of this gene is higher (>30), while in other groups, the threshold time of this gene is lower (<30). “B" means that the threshold time of this gene is relatively high (>30), that is, the threshold time of this gene is relatively high (>30), that is, the expression of this gene in the control group and the detection group is relatively small (that is, the expression of the gene in the control group and the detection group is relatively small), and the *p*-value of its crease variation is either unmeasurable or relatively large (*p* > 0.05). On the other hand, ‘C ′means that the average threshold time of the gene cannot be set, or that the threshold time exceeds the set critical time in both groups of samples (that is, the expression of the gene has not been found. The conclusion that the folding change is false and incomprehensible).

### 4.7 PD regulates key proteins of FXR-ASBT pathway in UC mice

**FIGURE 8:**
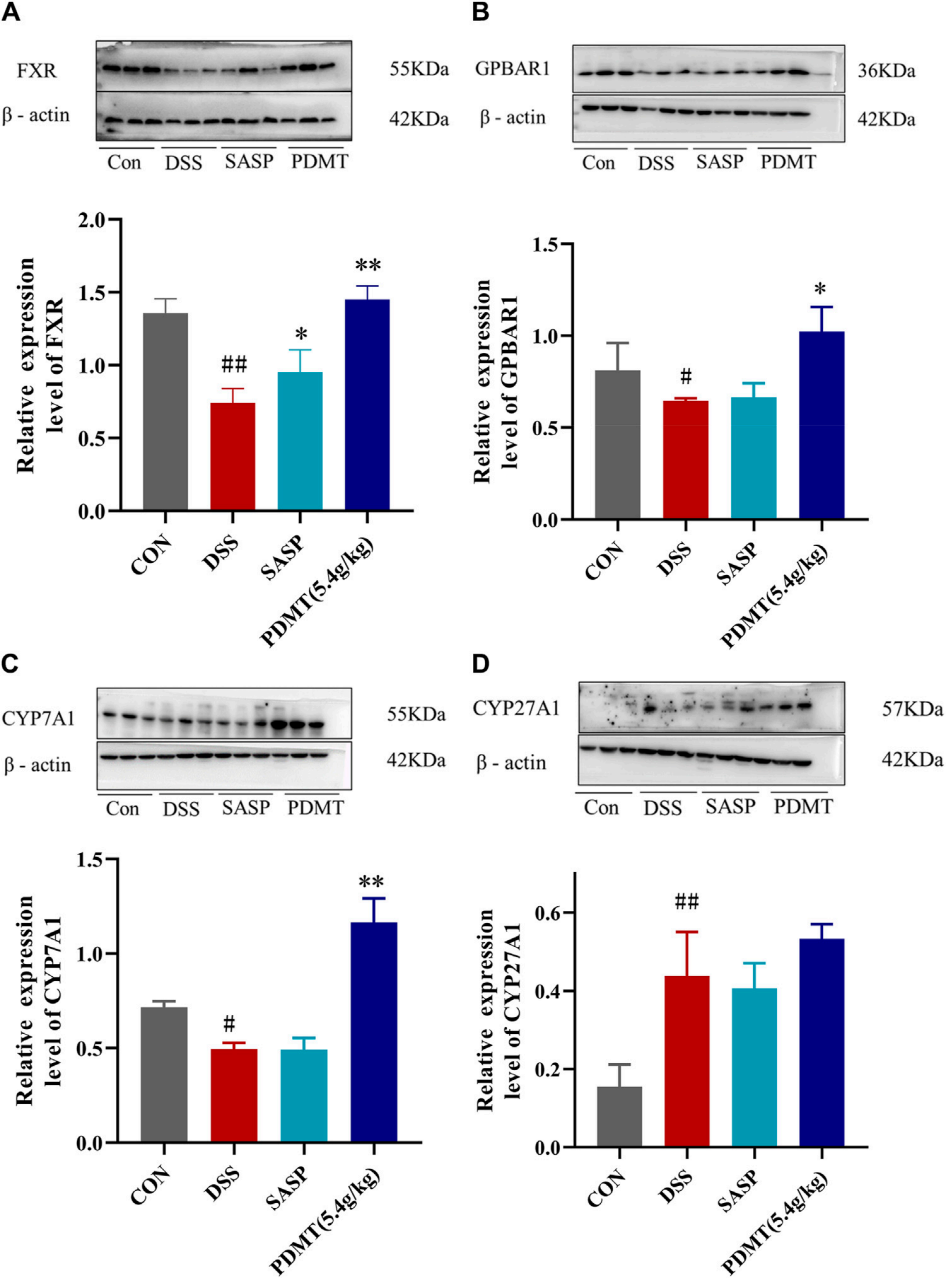
The changes of relative expression about FXR-ASBT pathway key proteins. **(A–D)** Relative expression of FXR, GPBAR1, CYP7A1, and CYP27A1 proteins in each group of mice colonic tissues. Data are shown as mean ± SEM (*n* = 3), #*p* < 0.05, #*p* < 0.05, ##*p* < 0.01 Compared with CON, *p < 0.05, **p < 0.01 Compared with DSS.

**FIGURE 9:**
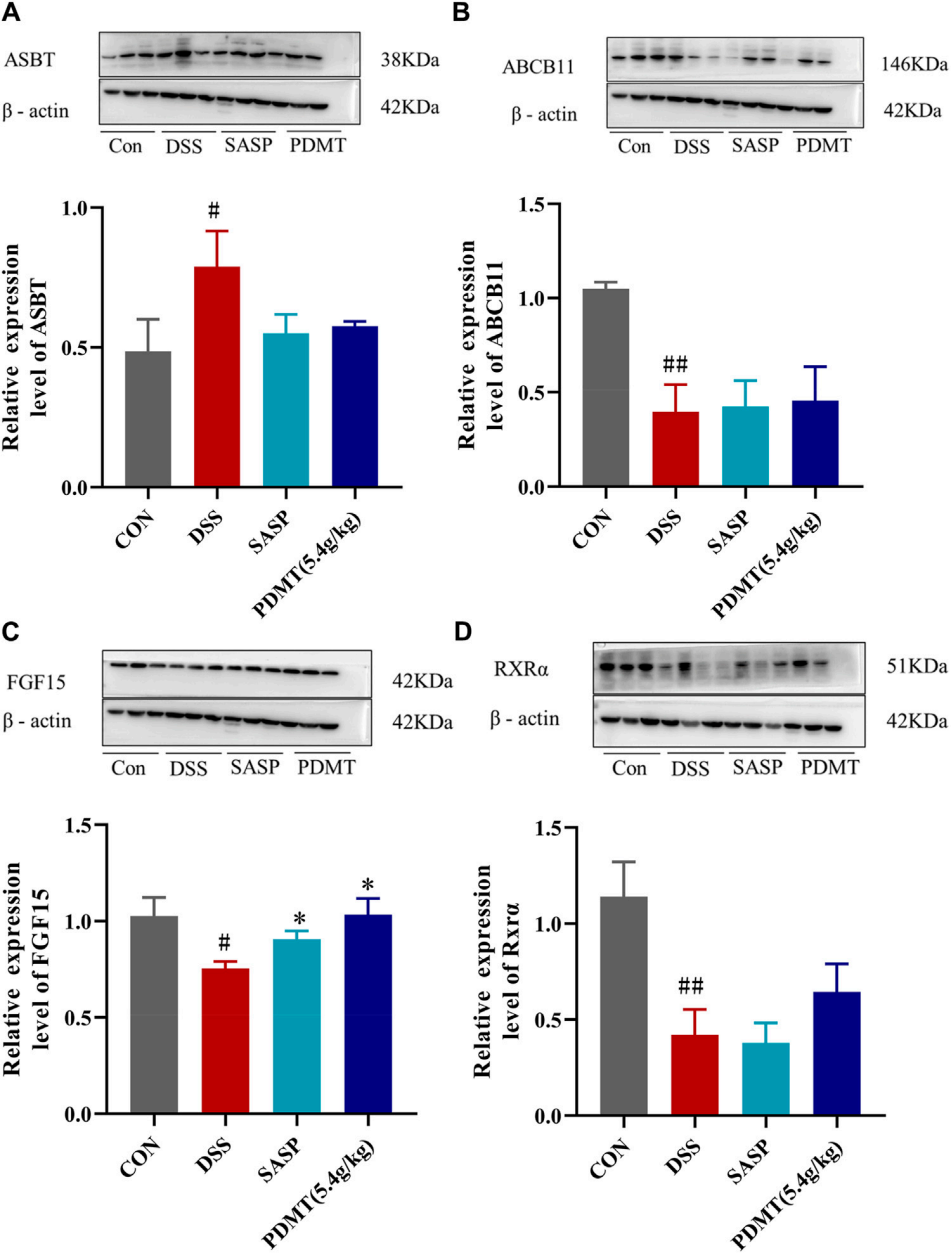
The changes of relative expression about FXR-ASBT pathway key proteins. **(A–D)** Relative expression of ASBT, ABCB11, FGF15, and RXRα proteins in each group of mice colonic tissues. Data are shown as mean ± SD (*n* = 3), #*p* < 0.05, ##*p* < 0.01 Compared with CON, *p < 0.05, **p < 0.01 Compared with DSS.

The protein expression levels of upstream and downstream marker genes of FXR-ASBT were validated through Western blot analysis. In comparison to the CON group, the relative expressions of FXR, TGR5, CYP7A1, ABCB11, FGF15, and RXRα proteins in colon tissues were significantly downregulated in the DSS group. Moreover, PD treatment significantly upregulated the relative expressions of FXR, TGR5, CYP7A1, and FGF15 proteins while showing a tendency to increase the relative expressions of ABCB11 and RXRα proteins (Figures 8A–C, 9B–D). Additionally, compared to the CON group, there was a significant upregulation in the relative expressions of CYP27A1 and ASBT proteins in mouse colon tissues within the DSS group; however, PDMT treatment tended to downregulate their relative expressions (Figures 8D, 9A).

## 5 Discussion

PD is a traditional prescription for frequent diarrhea and hematochezia. Our previous studies have shown that PD effectively inhibits DSS-induced UC by down-regulating inflammatory cytokines, regulating bile acids (BAs), and restoring intestinal flora (li Hua et al., 2021). To further elucidate the involvement of receptors in the bile acid pathway during the development of UC, we investigated the effect of PD on the expression of upstream and downstream genes related to bile acid receptors FXR and ASBT in mice. A mice model of UC was established and induced by 3.5% DSS, and then PCR array analysis was used to screen the differentially expressed genes related to FXR pathway in the colon tissues after PD treatment. After PD treatment, severe hematostool, weight loss, significantly shortened colon length, increased DAI score, and histopathological changes including goblet cell loss were improved to varying degrees, indicating that it has a good therapeutic effect on DSS-induced UC.

PCR array analysis was performed on 88 genes involved in the above pathways to find differentially expressed genes with significant changes.Genome-wide association studies and functional characteristics suggest that impaired bile acid signaling pathways may play a role in the development of inflammatory bowel disease (Fiorucci et al., 2021a). As shown in results, 42 genes from 88 bile acid related pathway were selected about PD treatment UC, 13 genes were upregulated and 29 genes were downregulated. These genes can be related to bile acid homeostasis (FXR, Abcc2, Abcc3, Abcb4, Abcb11, Abcg8, Slc10a1, OATPB3/8, Abcb11, Abcb4, Cyp7A1, Cyp8B1, CYP27A1, Cyp17a1, ASBT, OSTα-OSTβ), gut barrier function (ASBT, OSTα, OSTβ, GPBAR1,fxr,tgf15, Cyp2c70), and Intestinal inflammation (fxr, Tnf, Il10, Il23a, Il17a, Il6, Il1b, Il4).

FXR has a central role in bile acid homeostasis (Perino and Schoonjans, 2022). It maintains BAs homeostasis by controlling the synthesis, transport and metabolism of BAs (Sun et al., 2021). Active transporters of the ABC (ATP Binding Cassette) family are primarily responsible for drug efflux into the portal vein or into the bile. Abcc2, Abcc3, Abcb4, Abcb11, Abcg8, all belong to ABC (ATP Binding Cassette) family and Slc10a1, OATPB3/8, Abcb11, Abcb4 can participate in bile acids transport. Intestinal-hepatic circulation is an important way to maintain bile acid homeostasis. The bile salt export pump (BSEP/Abcb11) transports bile acids into the intestine, which is absorbed by ASBT into the intestinal epithelial cells in the ileum, and enters the portal vein through the heterodimerization of OSTα/β, and returns to the liver tissue (Gerloff et al., 1998; Liu et al., 2022). Major substrates of Abcb11 are taurine- and glycine-conjugated monovalent bile salts (Wang et al., 2022). In addition, enterohepatic circulation can recycle 95% of bile salts (Jia et al., 2021). In the result, the level of Abcb11 was decreased in DSS-reduce UC and PD can increase the level of Abcb11. Therefore, PD can interact with ABC target and increased the levels of Abcb11, impact bile acid export, thus maintaining bile acid homeostasis. Cyp7A1, Cyp8B1, CYP27A1, Cyp17a1 are BA synthesis enzymes, deficient of which disturb BA homeostasis (Guan et al., 2022). BAs closely regulate both CYP7A1 and CYP8B1 via feedback repression mediated by FXR-dependent stimulation of fibroblast growth FGF15/19 in the intestines (Gadaleta and Moschetta, 2019). The downregulation of CYP7A1 mRNA in liver can be achieved by binding FGF15/19 to FGF receptor 4/β-Klotho complex in hepatocytes (Jiang et al., 2021). In animal models, activation of FXR leads to decreased bile acid uptake by downregulating the expression of ASBT in intestinal epithelial cells (Tiratterra et al., 2018). ASBT, as a transporter of ileal bile acid reabsorption, is a key hub for intestinal bile acid-cholesterol balance and enterohepatic circulation bile acid (Yang et al., 2020). Loss of either the ASBT, OSTα-OSTβ transporters impairs intestinal bile acid absorption, characterization of the ASBT and Ostα-Ostβ null mice is beginning to reveal important phenotypic differences in bile acid homeostasis that could affect lipid and glucose metabolism (Huang et al., 2019). In the results, the level of ASBT, Ostα was increased and Ostβ was decreased, in DSS-reduce UC and PD can decrease the level of ASBT and Ostα. Therefore, PD can restore the level of ASBT, Ostα and Ostβ to repair the impairing intestinal bile acid absorption.

The biological barrier of the intestine is crucial for the maintenance of normal function. FXR and GPBAR1 (TGR5) play a key role in BAs-regulated intestinal barrier function (Fiorucci et al., 2023). *In vivo* and *in vitro* experiments in mice showed that the intestinal permeability was improved after treatment with FXR agonists (Gadaleta et al., 2011a). It was also found that after knockout of mouse GPBAR1 gene, the tight junction structure was destroyed and intestinal permeability increased (Cipriani et al., 2011). Targeting FXR and TGR5 signaling pathways may play an important role in restoring normal enterohepatic circulation and controlling UC inflammation. TGR5 agonists have shown promise in controlling inflammation in human cell models and animal models of IBD and UC (Jia et al., 2018). Osta-Ostb also appears to play a major role in protecting gut barrier injury (Ferrebee et al., 2018). The expression imbalance of ASBT and OSTa-OSTb is closely related to intestinal cell injury (Lun et al., 2024). The study also confirmed that the imbalanced BA–TGR5 axis could promote colonic mucosal barrier dysfunction and enhance visceral hypersensitivity (VH) in UC (Zhan et al., 2024). The expression of Cyp2c70 and FGF15 have closed relationship with impaired gut barrier (Hasan et al., 2023). In the result, the GPBAR1(TGR5) and FXR were increased treatment by PD and the expression levels of Occludin, Claudin1, Claudin5 and Zonula occludens-1 (ZO-1) protein, and the relative expression of ZO-1 and Occludin mRNA, which also related with colonic mucosal barrier, were significantly increased PD group in our previous study (Niu et al., 2023). PD can active the FXR and TGR5 to restore barrier impairment induced by DSS.

Intestinal inflammation strongly reduces FXR activation, probably via NF-κB-dependent tethering of FXR (Tang et al., 2022). FXR not only inhibits inflammation, but also is targeted by the inflammatory response itself (Feng et al., 2021). This could result in a vicious cycle where reduced FXR activity results in less repression of inflammation, contributing to development of chronic intestinal inflammation (Gadaleta et al., 2011b). Proinflammatory cytokines can cause intestinal barrier damage (Nunes et al., 2019). IL-1β can significantly increase the permeability of intestinal barrier, which depends on the activation of NF-κB (Zhong et al., 2021). Kaijia Tang found that Rc could reduce intestinal inflammation by activating FXR, resulting in NF-κB inhibition (Tang et al., 2022). It is well known that NF-κB is also a mediator of the intestinal barrier and regulates a variety of cellular signaling pathways (Yu et al., 2020). Inflammatory response is a characteristic of UC intestinal injury, which can promote its occurrence and development (Eichele and Kharbanda, 2017). Among the cytokines involved in UC pathology, IL-1β and IL − 23 are currently targeted drugs for UC treatment (Nakase et al., 2022). The imbalance of pro-inflammatory factors/anti-inflammatory factors in the colon and serum of the DSS group in this trial caused an inflammatory response in the intestine, which was restored by PD treatment. Levels of UC-related cytokines may vary depending on the stage of the disease (Nakase et al., 2022). Therefore, the serum IL-17 showed the opposite trend. The above results suggest that PD can improve intestinal inflammation and achieve therapeutic effects by regulating the levels of inflammatory factors in mice, which further activate the anti-inflammatory mechanism in mice.

**FIGURE 10:**
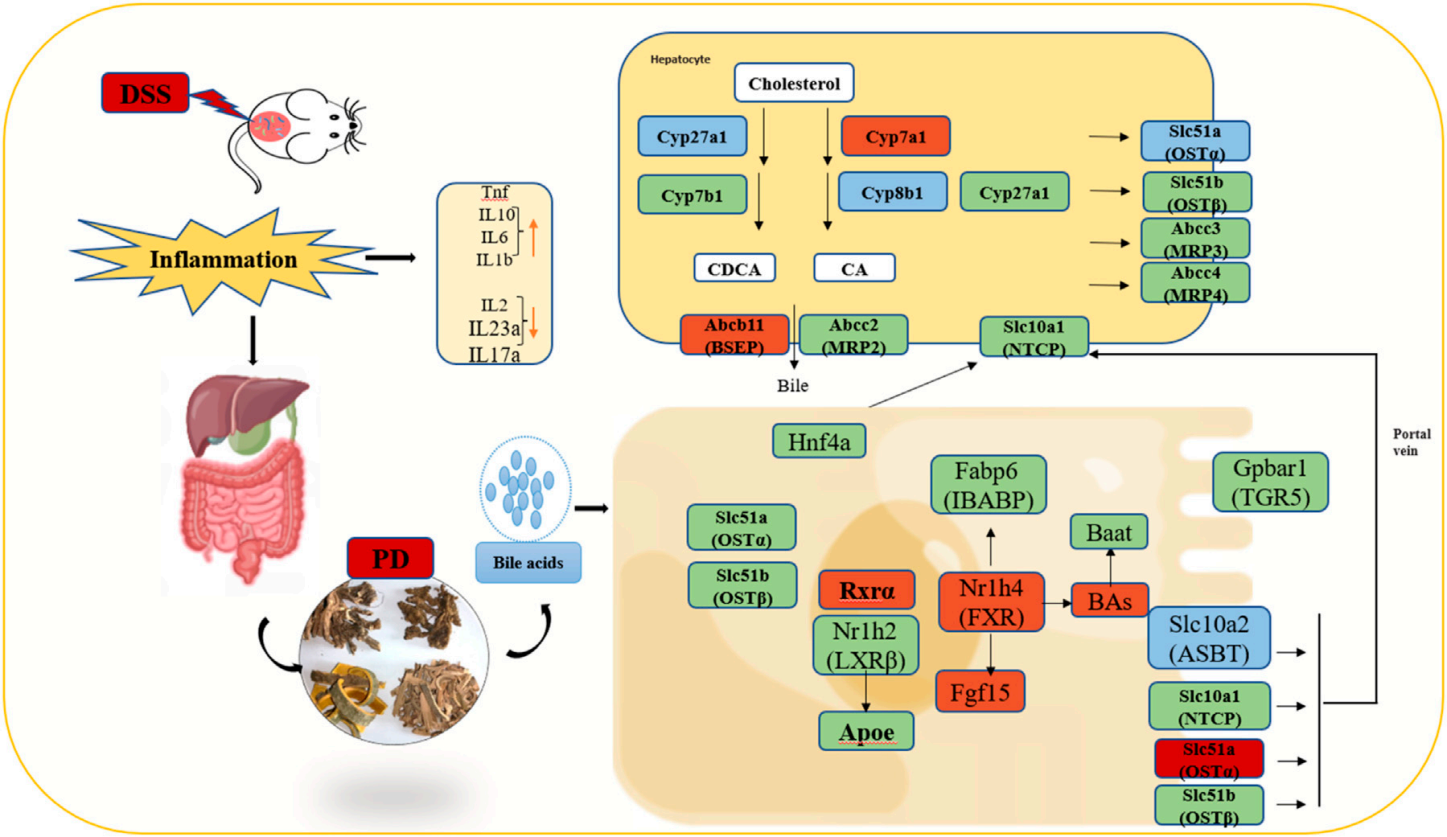
Illustration of the molecular mechanism of PD in treating colitis in mice. Note: Green color: having significant change genes after in DSS group; Blue color: having significant decreased genes after PD treatment; Red color: having significant increased genes after PD treatment; involved in the gut-liver axis of bile acid receptor in ulcerative colitis (UC).

Therefore, we can preliminarily propose the potential therapeutic mechanism of PD to treat UC in mice as follows: first, PD reinstated the BA homeostasis, which is mainly regulated by FXR and ABC family genes. Moreover, it regulated BA receptor, which further induced the activation of FXR and TGR5, inhibit ASBT to greatly improve gut barrier function and reduce intestinal inflammation Figure 10.

This study has two limitations. First, while the west blot analysis and qPCR results in our study showed activation of the FXR-ASBT gene in PD-treated colitis mice, which indicated that regulatory mechanism may exist for FXR-ASBT. However, we have not identified the specific underlying mechanisms involved FXR, given the multitude of regulatory signals influencing FXR. Second, there were a variety of bile acids that are changed after PD in DSS-treated mice in liver, but we focus on the role of colon. Therefore, we did not perform research on genes of liver that deserve deeper investigation.

## 6 Conclusion

PD can alleviate the tissue damage and inflammatory symptoms of DSS-induced colitis in mice, and has a good therapeutic effect on it. The mechanism may be related to inhibiting inflammation and regulating bile acid metabolism by regulating FXR-ASBT signaling pathway. It provides an important theoretical basis for the further development of PD in the treatment of UC.

## Data Availability

The datasets presented in this study can be found in online repositories. The names of the repository/repositories and accession number(s) can be found in the article/Supplementary Material.
