# Supplementary material for: Pulsatilla decoction alleviates DSS-induced UC by activating FXR-ASBT pathways to ameliorate disordered bile acids homeostasis
Source: Front Pharmacol. 2024 Jun 21;15:1399829. doi: 10.3389/fphar.2024.1399829 (PMC11224520; doi:10.3389/fphar.2024.1399829)
Supplement: Supplementary file 1 [file DataSheet1.docx]

**Reference description:**

**Based on the results of the previous research of our group, the main chemical components in PD were detected by HPLC.** **The specific results are as follows:**

**Determination of 6 index components in Pulsatilla decoction by HPLC**

1Result

1.1 Method Specificity

The results are shown in Fig.1.Under the mixed standard chromatographic conditions, the five main components ( aesculin, aesculetin, jatrorrhizine, palmatine, berberine hydrochloride ) in the high performance liquid chromatogram of Pulsatilla decoction were well separated from the chromatographic peaks of other components, and the retention time was consistent with the mixed standard reference substance. The retention time of aesculin, aesculetin, jatrorrhizine, palmatine and berberine hydrochloride were 3.356,5.739,14.447,17.677 and 18.135, respectively. Compared with the chromatogram of Pulsatilla decoction, the chromatograms of the negative control solution lacking Cortex Fraxini and the negative control solution lacking Rhizoma Coptidis and Cortex Phellodendri were significantly different, indicating that the method had good specificity.
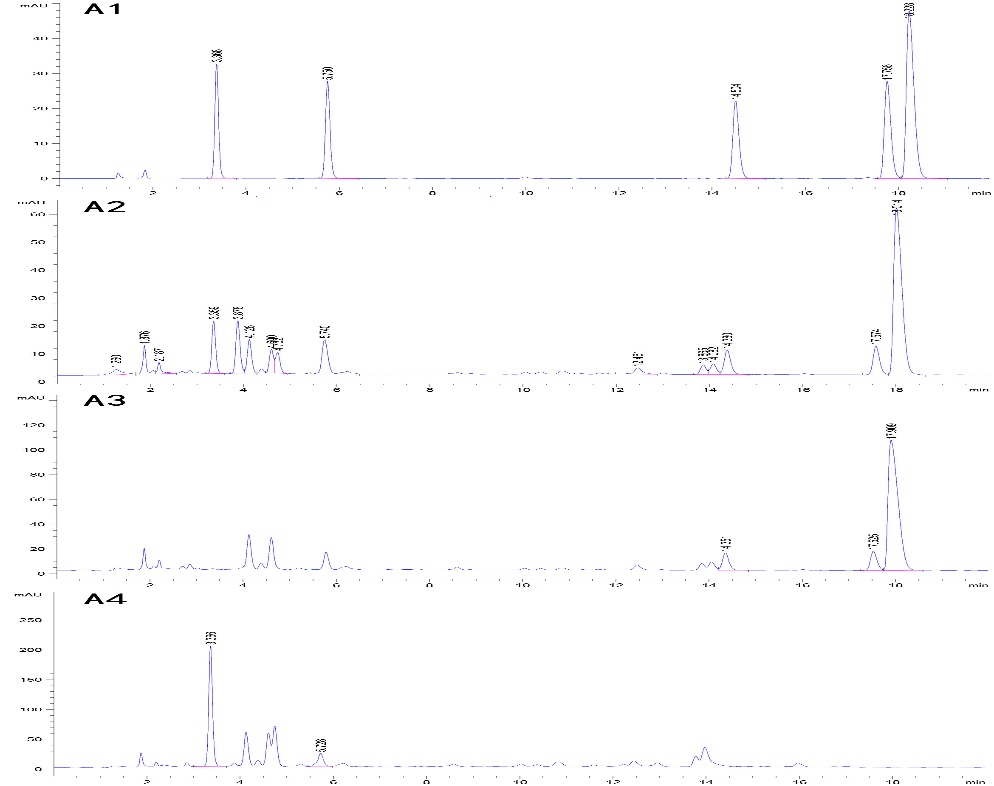


Figure1. HPLC chromatograms of reference substances and samples.

Note: A1: Reference substances by HPLC-UN (335 nm); A2: Samples by HPLC- UN (335 nm); A3: Sample of lacking fraxini cortex by by HPLC-UN (335 nm); A4: Sample of lacking coptidis rhizome, phellodendri cortex by HPLC-UN (335 nm)

As shown in Fig.2, under the chromatographic conditions of Pulsatilla saponin B4, the chromatographic peaks of Pulsatilla saponin B4 in the high performance liquid chromatogram of Pulsatilla decoction were well separated and consistent with the retention time of the mixed standard reference substance. Compared with the chromatogram of Pulsatilla decoction, the chromatogram of negative control solution without Pulsatilla was significantly different, indicating that the method had good specificity.


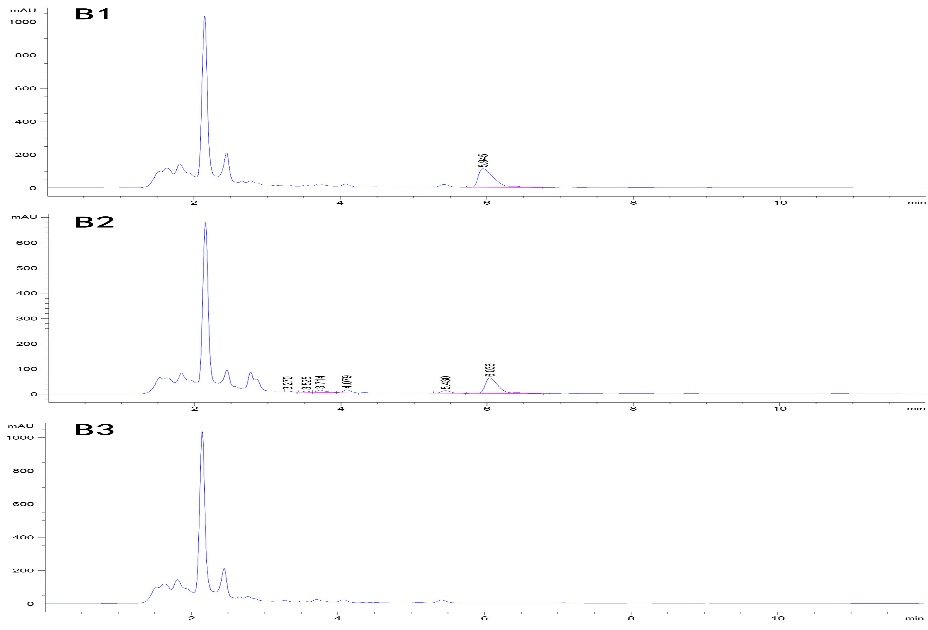


Figure2. HPLC chromatograms of reference substances and samples.

Note: B1: Reference substances by HPLC-UN (205 nm); B2: Samples by HPLC- UN (205 nm); B3: Sample of lacking pulsatillae radix by by HPLC-UN (205 nm)

3.2 Linear relationship and quantitative limit

The results are shown in Table 1, and the r2 of the six main chemical components was greater than 0.99, indicating that the components had good linearity in the linear range.

**Table 1 Regression equations of reference substances**

| Reference substance | Regression equation | Linearity range/μg·mL−1 | r^2^ |
| --- | --- | --- | --- |
| Aseculin | Y=44.2726X+0.2575 | 1.04~6.24 | 0.9999 |
| Aesculetin | Y=51.067X-0.1568 | 0.92~5.52 | 0.9998 |
| Jatrorrhizine | Y=74.2331X-2.7661 | 0.7~4.2 | 0.9987 |
| Palmatine | Y=67.3416X-4.6761 | 1.04~6.24 | 0.9978 |
| Berberine hydrochloride | Y=62.7494X-5.6567 | 2.2~13.2 | 0.9979 |
| Pulchinenoside B4 | Y=4.3349X+3.080 | 5.5~33 | 0.998 |

3.3 Precision test

The results of intra-day precision showed that the RSD of aesculin peak area was 0.49 %, the RSD of aesculetin peak area was 0.41 %, the RSD of jatrorrhizine peak area was 1.22 %, the RSD of palmatine peak area was 1.05 %, the RSD of berberine hydrochloride peak area was 1.20 %, and the RSD of anemoside B4 peak area was 0.27 %. The RSD of precision test was less than 2 %, indicating that the instrument had good precision.

The results of precision showed that the peak area RSD of aesculin, aesculetin, jatrorrhizine, palmatine, berberine and anemoside B4 were 0.54 %, 0.48 %, 1.09 %, 0.95 %, 1.23 % and 0.21 %, respectively, indicating that the precision of the instrument was good.

3.4 Replica test

The results showed that the RSDs of peak area of aesculin, aesculetin, jatrorrhizine, palmatine, berberine and anemoside B4 were 0.34 %, 0.67 %, 0.95 %, 1.12 %, 0.53 % and 0.23 %, respectively, indicating that the method had good repeatability.

3.5 Stability test

The RSD of the peak area of the six components was 1.51 %, 1.36 %, 1.94 %, 1.22 %, 1.23 %, and 1.47 % ( n = 6 ), respectively, which indicated that the test solution of Pulsatilla decoction was stable within 12 hours at room temperature.

3.6 Sample recovery

The results showed that the recoveries of aesculin, aesculetin, jatrorrhizine, palmatine, berberine and anemoside B4 were 98.74 % ( RSD = 1.01 % ), 98.12 % ( RSD = 0.36 % ), 97.90 % ( RSD = 1.32 % ), 96.95 % ( RSD = 1.68 % ), 97.43 % ( RSD = 1.80 % ) and 98.45 % ( RSD = 1.16 % ), respectively, indicating that the established method had good accuracy.

3.7 Determination of the content of sample

As shown in Table 2, the contents of aesculin, aesculetin, jatrorrhizine, palmatine, berberine and anemoside B4 in Pulsatilla decoction were 0.7 %, 0.36 %, 0.12 %, 0.38 %, 8.26 % and 4.71 %, respectively.

**Table 2. The main components contents in PD**

| PD | Aesculin | Esculetin | Jateorhizine | Palmatine | Berberine | Anemoside B4 |
| --- | --- | --- | --- | --- | --- | --- |
| 1 kg Crude herb | 43.98mg | 31.13mg | 10.54mg | 15.31mg | 108.29mg | 232.95mg |

Note: The main components contents of PD extract were measured by HPLC method as described in Section 2. The value given is the equivalent amount of the component present in the indicated amount (dose) of PD extract used for animal treatment. The italicized number in parentheses is the percent.
